# Association of P2X7 polymorphisms on Type 2 diabetes mellitus susceptibility and diabetic complications

**DOI:** 10.1371/journal.pone.0318134

**Published:** 2025-01-27

**Authors:** Guoni Huang, Jing Cheng, Wenfeng Liu, Tong Yang, Tao Ye, Qian Zhang, Qi Chen, Yuzhong Xu

**Affiliations:** 1 Department of Laboratory Medicine, People’s Hospital of Shenzhen Baoan District, Shenzhen, P. R. China; 2 State Key Laboratory of Respiratory Disease, Guangdong Provincial Key Laboratory of Protein Modification and Degradation, School of Basic Medical Sciences, Guangzhou Medical University, Guangzhou, P. R. China; 3 Department of Endocrine Medicine, People’s Hospital of Shenzhen Baoan District, Shenzhen, P. R. China; Kerman University of Medical Sciences Physiology Research Center, ISLAMIC REPUBLIC OF IRAN

## Abstract

**Objectives:**

This case-control study aims to clarify the impact of single nucleotide polymorphisms (SNPs) within the P2X7 gene on susceptibility to type 2 diabetes mellitus (T2DM) and to evaluate their association with diabetic complications.

**Methods:**

This study is comprised with 200 T2DM cases and 200 healthy controls. Seven candidate SNP loci were screened, and TaqMan-MGB real-time PCR technology was used to determine the polymorphic variants of P2X7. Different genotype and allele frequencies were compared by Pearson’s χ2 tests and logistic regression analysis.

**Results:**

Three P2X7 SNPs were found to be associated with T2DM risk. Specifically, rs7958311 GA (OR = 1.323, *p* = 0.002), rs7958311 AA (OR = 1.508, *p* = 0.038), rs208294 CC (OR = 1.854, *p* = 0.042) showed a higher susceptibility to T2DM, whilst rs11065464 CA (OR = 0.614, *p* = 0.022) was associated with a reduced risk. Logistic regression analysis indicated that rs7958311 was linked to an increased risk for nephropathy (OR = 1.833, *p* = 0.022), but with a decreased risk for peripheral artery disease (OR = 0.550, *p* = 0.042). Additionally, rs208294 was identified as a risk factor for peripheral neuropathy (OR = 2.101, *p* = 0.016).

**Conclusions:**

We found that P2X7 polymorphisms are significantly associated with the risk of T2DM and its complications, suggesting that targeting P2X7 may offer a novel therapeutic strategy for the prevention and personal treatment of T2DM.

## Introduction

Type 2 diabetes mellitus (T2DM) is a chronic metabolic disorder characterized by hyperglycemia, arising from a complex interplay of genetic, lifestyle, and environmental factors [1,2]. This multifactorial and polygenic disease often leads to severe complications, including nephropathy, retinopathy, peripheral neuropathy, coronary artery disease (CAD), peripheral artery disease(PAD) and ischemic stroke [3]. Notably, twin family studies have demonstrated significant variability in complication rates among individuals, particularly between those with a family history of diabetes-related complications and those without [4,5]. These observations highlight the potential of genetic analysis to predict individual risks of developing T2DM and its associated complications, therefore to achieve precision medicine for individuals.

To date, numerous risk loci influencing T2DM susceptibility and its complications have been identified, yet many remain undiscovered [6]. Among these, the Purinergic P2X7 receptor (P2X7R), an eATP-gated ionic channel expressed in various tissues, has emerged as a significant candidate [7]. Multiple studies have demonstrated that P2X7R plays a regulatory role in pancreatic β-cell proliferation, insulin secretion and involvement in T2DM pathogenesis [8,9]. Furthermore, given that inflammation is a major mechanism of tissue and organ damage, is also influenced by P2X7R, extends to several T2DM complications [10,11].

The P2X7R gene is highly polymorphic, with numerous single nucleotide polymorphisms (SNPs) affecting its expression and function [9,12]. A genome-wide association study (GWAS) has identified genomic regions involved in T2DM pathogenesis, and these polymorphisms may increase the risk of developing T2DM [13]. However, the specific association between P2X7 polymorphisms and T2DM susceptibility has yet to be documented. Consequently, this study aims to investigate the association of P2X7 SNPs with T2DM susceptibility and the development of diabetic complications. Our objective is to fill the existing gaps in knowledge and contribute to the development of targeted interventions that could mitigate the onset and progression of T2DM.

## Methods

### Study subject and eligibility

This study included 200 T2DM patients and 200 healthy controls, recruited from the People’s Hospital of Shenzhen Baoan District between March 2022 to September 2023. The T2DM patients were diagnosed with fasting plasma glucose levels ≥7.0 mmol/L in accordance with the 1999 WHO diagnostic criteria in [14]. Exclusion criteria included patients with other types of diabetes, malignancies, other endocrine diseases or chronic conditions. Specifically, the exclusion criteria for chronic diseases focused on patients with severe chronic kidney disease (CKD) and significant heart failure. For CKD, patients with an estimated Glomerular Filtration Rate (eGFR) below 30 mL/min/1.73 m^2^ were excluded. This threshold is in line with the guidelines for stage 4 CKD, aimed at excluding individuals with advanced renal impairment. For heart Failure, patients with an ejection fraction (EF) of less than 35% were also excluded. This cutoff was used to identify patients with severe systolic dysfunction, thereby minimizing confounding due to advanced cardiac conditions. The healthy controls had no family or personal history of diabetes and were free from any current health issues. Clinical characteristics such as age, gender, height, weight, smoking status, drinking habits, blood pressure and complications were collected, with follow-up assessments conducted during the study. This work received approval from the ethics committee of Shenzhen Baoan District People’s Hospital (BYL20220223) and adhered to the Declaration of Helsinki. All patients signed written informed consents before participating in the study.

### SNP selection

Seven P2X7 SNPs (rs3751143, rs1718119, rs17525809, rs7958311, rs208294, rs2230911, rs11065464) were selected for genotyping from the HapMap project database (http://www.hapmap.org/) and NCBI database (https://www.ncbi.nlm.nih.gov/projects/SNP/).

SNPs were selected based on the following criteria:1) located within gene regions that may have functional effects, and 2) minor allele frequencies (MAFs) greater than 5% in the Chinese population.

### Sample collection and biochemical detection

Blood samples (5ml) were collected into serum and EDTA tubes from each participant. Genomic DNA was extracted from peripheral leukocytes using a Genomic DNA Extraction Kit (Tiangen Biotech, China), and its concentration was assessed using an ultraviolet spectrophotometer (UV-1900I, Shimadzu Scientific Instruments, Japan). The isolated DNA samples were stored at −20°C until analysis. Serum samples, obtained after centrifugation, were used for biochemical measurements, including fasting blood glucose (FBG), HbA1c, triglyceride (TG), total cholesterol(TC), high-density lipoprotein cholesterol(HDL-C) and low-density lipoprotein cholesterol(LDL-C).

### P2X7 genotyping

Primers and TaqMan-MGB probe sequences for P2X7 were designed based on the NCBI database, using the Primer Express3.0.1(Applied Biosystems, CA, USA) (S1 Table). Genotyping was conducted using TaqMan-MGB real-time PCR technology, with reagents prepared as follows: 10μL of 2×TaqMan genotyping master mix, 3 μM primers, 2 μM TaqMan-MGB probes, 20 ng DNA sample and sterile water for a total volume of 25 μL. PCR conditions were as follows:initial denaturation at 95°C for 5 minutes, followed by 45 cycles of denaturation at 95°C for 10 seconds and annealing at 60°C for 30 seconds.Amplifications and detection were performed using a LightCycler 480 II instrument (Roche, Basel, Switzerland). For quality control, 5% of DNA samples were randomly selected for DNA sequencing, and the results showed 100% repeatability.

### Statistical analysis

Statistical analyses was performed using SPSS software version 17.0(SPSS Inc, Chicago, USA). Clinical and biochemical data were presented as means ± SD (for quantitative data) or percentages (for categorical data). Categorical variables were compared using Pearson’s χ2 test, while quantitative data were analyzed using the independent-samples t test. The Hardy-Weinberg equilibrium (HWE) in the control group was assessed with Pearson’s χ2 test by comparing observed and expected frequencies. Genotypic and allelic frequencies in the case and control groups were compared using Pearson’s χ2 test, with odds ratios (OR) with 95% confidence intervals(CI). A logistic regression model was used to determine the association between P2X7 SNPs and the risk of diabetic complications. *p*<0.05 was considered statistically significant.

## Results

### Demographic data and biochemical parameters of the studied population

A total of 400 participants were enrolled in the study, comprising 200 individuals with type 2 diabetes mellitus (T2DM) and 200 healthy controls. Table 1 presents the demographic and biochemical characteristics of the subjects. The were no statistically significant differences in age,the number of drinkers, and HDL-C between the case and control groups (*p*>0.05). However, the means of males, body mass index(BMI), smokers, hypertension, FBG, HbA1c, TG, TC, LDL-C were significantly higher in T2DM cases compared to healthy controls(*p*<0.05).

**Table 1:** Demographic data and biochemical parameters between case and control groups.

Variables	Cases(n = 200)	Controls(n = 200)	*p* value
Age (mean ±SD)	53.09±13.66	52.38±4.77	0.526
Gender, Male/Female	136/64	108/92	**0.004**
BMI, kg/m^2^	26.45±5.09	23.15±4.06	**0.001**
Smoking, N(%)	79(40)	56(28)	**0.015**
Drinking, N(%)	38(19)	41(21)	0.802
Hypertension, N(%)	61(31)	38(19)	**0.011**
FBG (mmol/L)	11.39±5.19	5.19±0.73	**<0.001**
HbA1c(%)	9.58±2.57	4.98±0.44	**<0.001**
TG (mmol/L)	2.59±2.85	1.73±1.09	**0.006**
TC (mmol/L)	5.14±2.83	4.41±0.84	**0.019**
HDL-C (mmol/L)	1.22±0.37	1.35±0.32	0.068
LDL-C (mmol/L)	3.40±1.42	2.94±0.86	**0.012**
Nephropathy, N(%)	29(14.5)		
Retinopathy, N(%)	38(19)		
Peripheral neuropathy, N(%)	47(23.5)		
CAD, N(%)	28(14)		
PAD, N(%)	22(11)		
Ischemic stroke, N(%)	9(4.5)		

Notes: *p* values were calculated by Pearson’s χ^2^ test for categorical variables and Independent-samples t test for continuous variables; Bold values indicate that *p*<0.05 considered as statistical significance.

Abbreviation: BMI, body mass index; FBG, fasting blood glucose; TG, triglyceride; TC, total cholesterol; HDL-C, high-density lipoprotein cholesterol; LDL-C, low-density lipoprotein cholesterol; CAD, coronary artery disease; PAD, peripheral artery disease.

### Basic information of P2X7 SNPs

The call rate of rs3751143 was below 50% in both case and control groups, rendering it unsuitable for association analysis; therefore, this locus was excluded from further discussion. The call rates for the remaining SNPs were above 99%, with basic information presented in Table 2. All allele frequencies of the six SNPs were consistent with the Hardy-Weinberg equilibrium (HWE) in the control group (*p* > 0.05). Pearson’s chi-squared test was used to compare allele distributions between the case and control groups. Three P2X7 SNPs (rs7958311, OR = 1.448, *p*<0.001; rs208294, OR = 1.315, *p* = 0.005; rs11065464, OR = 0.734, *p =* 0.002) were found to be associated with T2DM risk, while the other SNPs showed no significant association with T2DM susceptibility.

**Table 2:** Information and allele frequencies of P2X7 candidate SNPs in this study.

SNP_S_ ID	Chr:Position	Allele A/B	Frequency (MAF) Case Control		HWE *p* value	OR (95% CI)	*p* value
rs1718119	12:121615103	G/A	0.109	0.116	0.268	0.932(0.706–1.230)	0.620
rs17525809	12:121592689	T/C	0.027	0.031	0.923	0.867(0.514–1.464)	0.594
rs7958311	12:121605355	G/A	0.468	0.378	0.757	**1.448(1.211–1.730)**	**<0.001**
rs208294	12:121600253	T/C	0.421	0.357	0.587	**1.315(1.093–1.564)**	**0.005**
rs2230911	12:121615131	C/G	0.144	0.136	0.549	1.069(0.830–1.376)	0.606
rs11065464	12:121602135	C/A	0.287	0.358	0.539	**0.734(0.603–0.895)**	**0.002**

Notes: *p* values were calculated by Pearson’s χ^2^ test; Bold values indicate that *p*<0.05 considered as statistical significance.

Abbreviation: SNP, single nucleotide polymorphism; Chr, chromosome; Allele A/B, Minor/Major allele; MAF, minor allele frequency; HWE, Hardy-Weinberg equilibrium; OR, odds ratio; CI, confidence interval.

### Association of the P2X7 SNPs with the risk of T2DM

We further analyzed the allele and genotype frequencies of three P2X7 SNPs between the case and control groups, with results shown in Table 3. The T2DM-susceptibility allele for rs7958311 was A (OR = 1.238, *p* = 0.012). Individuals with GA and AA genotypes exhibited a higher risk of T2DM (GA, OR = 1.323, *p* = 0.002; AA, OR = 1.508, *p* = 0.038). The dominant model (AA+GA vs GG) further indicated an increased risk associated with the A genotype (OR = 1.989, *p* = 0.002). The T2DM-susceptibility allele for re208294 was C (OR = 1.217, *p* = 0.046), with the CC genotype being a risk factor for T2DM (OR = 1.854, *p* = 0.042). Regarding rs11065464, the dominant model (AA+CA vs CC: OR = 0.614, p = 0.021) showed that the A genotype was associated with a reduced risk of T2DM (OR = 0.725, p = 0.041). Therefore, rs11065464 CA genotype (OR = 0.614, p = 0.022) is likely to be a protective factor against T2DM.

**Table 3:** Allele and genotype frequencies of three SNPs of P2X7 between case and control groups.

SNP_S_ ID	Models	Genotype	Cases (n = 200)	Controls (n = 200)	OR (95% CI)	*p* value
rs7958311	Allele	G	213	249	1	
		A	187	151	**1.238(1.051–1.459)**	**0.012**
	Genotype	GG	51	81	1	
		GA	111	87	**1.323(1.106–1.583)**	**0.002**
		AA	38	32	**1.508(1.032–2.204)**	**0.038**
	Dominant	AA+GA vs GG			**1.989(1.300–3.042)**	**0.002**
	Recessive	AA vs GA+GG			1.188(0.774–1.821)	0.511
rs208294	Allele	T	232	257	1	
		C	168	143	**1.217(1.009-1.468)**	**0.046**
	Genotype	TT	71	81	1	
		TC	90	95	1.081(0.703-1.664)	0.743
		CC	39	24	**1.854(1.017–2.359)**	**0.042**
	Dominant	CC+TC vs TT			1.414(0.951–2.102)	0.107
	Recessive	CC vs TC+TT			1.537(0.884–2.671)	0.164
rs11065464	Allele	C	285	257	1	
		A	115	143	**0.725(0.538-0.977)**	**0.041**
	Genotype	CC	101	77	1	
		CA	83	103	**0.614(0.406-0.930)**	**0.022**
		AA	16	20	0.610(0.297–1.254)	0.201
	Dominant	AA+CA vs CC			**0.614(0.412–0.913)**	**0.021**
	Recessive	AA vs CA+CC			0.783(0.393–1.558)	0.601

Notes: *p* values were calculated by Pearson’s χ^2^ test; Bold values indicate that *p* <0.05 considered as statistical significance.

### Analysis of the association between P2X7 SNPs and T2DM complications

Among the 200 T2DM patients studied, 114 had microvascular complications (14.5% nephropathy, 19% retinopathy and 23.5% peripheral neuropathy), and 59 had macrovascular complications (14% CAD, 11% PAD and 4.5% ischemic stroke) (see Table 1).

**Fig 1:**
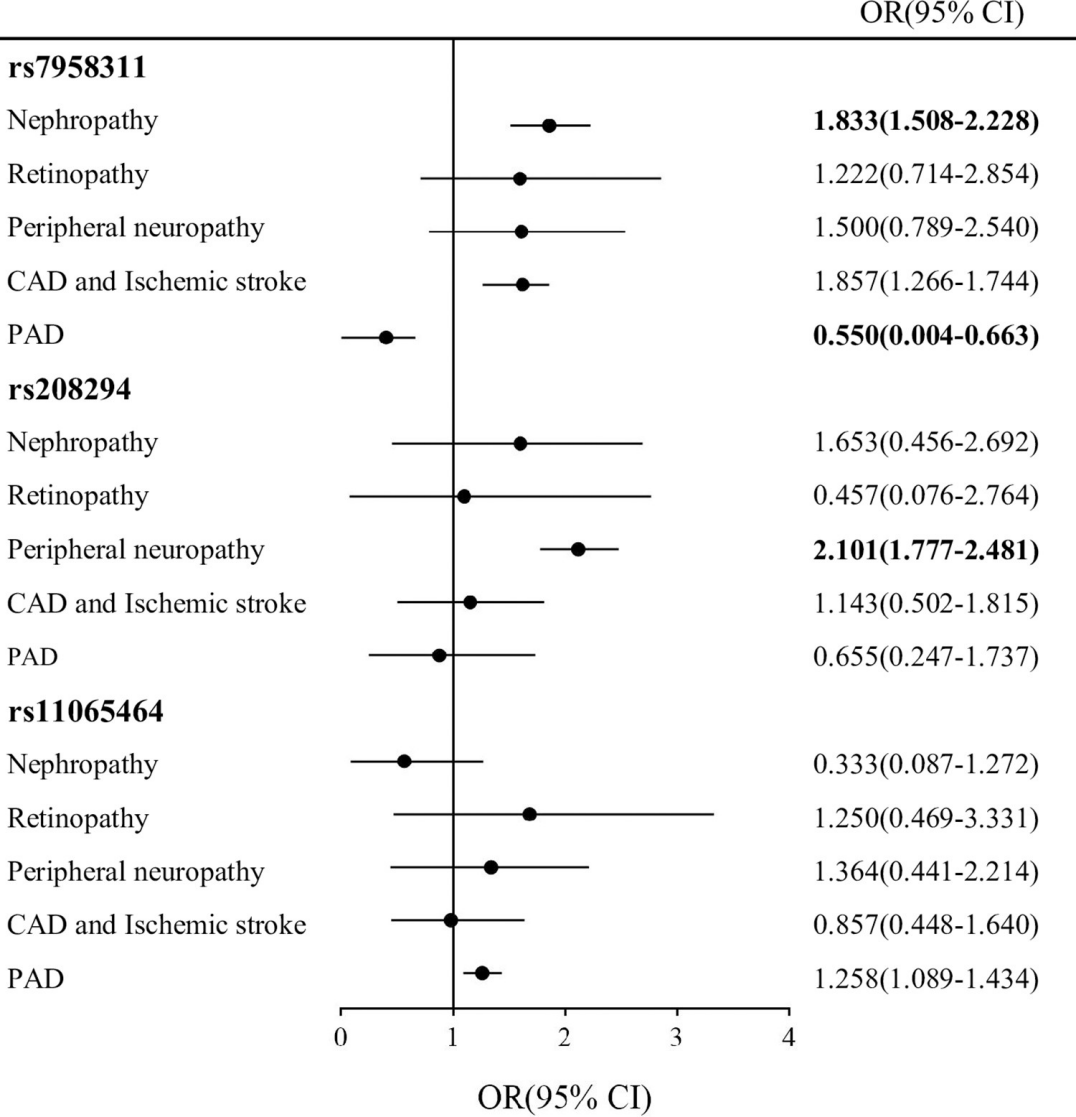
Effect of three P2X7 SNPs on the risk of diabetic complications in T2DM patients. Notes: *p* values were calculated by logistic regression analysis and expressed OR (95% CI), adjusted for age, gender, BMI, Smoking, Hypertension. Bold values indicate that *p* <0.05 considered as statistical significance. Abbreviation: OR, odds ratio; 95% CI, 95% confidence interval; CAD, coronary artery disease; PAD, peripheral artery disease.

Based on previous studies, we investigated the association between three P2X7 SNPs and the risk of complications in T2DM patients. In a logistic regression model, adjusting for factors such as age, gender, BMI, smoking and hypertension, we examined the three P2X7 SNPs as independent variables. Fig 1 presents the results of genetic risk associated with various complications in the case group. We found that rs7978311 significantly increased the risk of nephropathy (OR = 1.833, *p* = 0.022) while exerting a protective effect against PAD in T2DM patients (OR = 0.550, *p* = 0.042). Additionally, rs208294 was associated with an increased risk of peripheral neuropathy in T2DM patients (OR = 2.101, *p* = 0.016). In contrast, rs11065464 showed no significant association with these complications.

## Discussion

For the first time, this study explored the potential effect of P2X7 polymorphism on T2DM susceptibility and complications. The demographic data and biochemical parameters revealed that gender, BMI, smoking status, hypertension, FBG, HbA1c, TG, TC, LDL-C were associated with an increased risk of T2DM. Additionally, we screened seven P2X7 SNPs using a database. Notably, the call rate of rs3751143 was low in both the case and control groups.It may be due to technical challenges in genotyping this specific SNP, such as suboptimal DNA quality or inherent limitations in assay design. In contrast, the remaining SNPs exhibited high call rates and were subjected to an in-depth and comprehensive analysis. These SNPs furnished reliable and valuable insights into the potential association between P2X7 polymorphisms and the risk of T2DM. Among them, rs7958311, rs208294 and rs11065464 were found to be associated with T2DM risk, thereby warranting further investigation and discussion in this research.

For SNP rs7958311, the GA and AA genotypes were associated with an increased risk of T2DM. Furthermore, rs7958311 also significantly contributed to the risk of nephropathy while showing a protective effect against PAD in T2DM patients.

Previous reports indicate that the rs7958311 coding SNP leads to an amino acid change from arginine to histidine (Arg270>His) in an extracellular subunit of the P2X7 transmembrane receptor, resulting in a gain of function [15,16]. Todd et al. found that rs7958311 was associated with higher 2-hour glucose and 2-hour proinsulin levels in a mouse model [9]. In humans, this is the first study showing a significant association between rs7958311 and the risk of T2DM. Moreover, studies have shown that P2X7 gene might associated with the risk and prognosis of human tuberculosis, where each additional A allele of SNP rs7958311 increased the likelihood of successful treatment outcomes by 59% [17]. Therefore, understanding the role of SNP rs7958311 in T2DM could thus contribute to the development of new treatment targets.

For SNP rs208294, the CC genotype was associated with increased risk of T2DM, and was a risk factor for peripheral neuropathy in T2DM patients. The SNP rs208294 is the most frequent human P2X7 variant (with a minor allele frequency of 40% in Asians), leading to the substitution of histidine 155 to tyrosine(His155Tyr) [18]. This mutation may increase the level of P2X7 receptor expression on the cell surface, potentially leading to altered immune responses and increased susceptibility to metabolic stress [19]. For example, Pegoraro et al. revealed that due to the increased cell surface expression of P2X7 receptor by SNP rs208294, enhanced the virus-cell interaction, thereby facilitating viral infections [20]. Further work is needed todetermine how rs208294 affects T2DM risk by altering P2X7 receptor function.

For SNP rs11065464, the CA genotype was associated with a decreased risk of T2DM, though it was not significantly linked to any complications. Previous studies have found that the minor allele (A) of rs11065464 is associated with elevated fasting proinsulin levels, which may be the mechanism by which rs11065464 could mitigate T2DM risk [9]. But currently there is limited research on rs11065464, and large sample sizes are necessary to validate these associations.

The association between P2X7 polymorphisms and T2DM risk, as well as its complications, suggests that these genetic markers could potentially be used for early risk assessment in clinical practice. Identifying individuals with high-risk genotypes may allow for personalized prevention strategies and more targeted treatments for T2DM and its complications.

### Limitations and strengths

While several parameters did not show statistically significant differences, their inclusion in the study is crucial for a few reasons. First, these parameters are often associated with the conditions under investigation in prior research, which are well-known risk factors for T2DM and its complications, thus their analysis helps in comprehensively understanding the disease framework and validates our study against existing literature. Second, understanding the absence of significance in these variables can help to clarify the focus on those parameters that are truly associated with T2DM risk and progression. There are, however, some limitations to our study. First, the sample size is relatively small, and future studies should include larger samples from different geographical regions and ethnic groups to validate these findings. Second, the potential mechanisms through which P2X7 SNPs influence T2DM risk are not fully understood. Future studies should investigate these SNPs further to clarify their role in inflammatory pathways and how they may modulate the risk of T2DM. Going forward, we plan to investigate the association between P2X7 and T2DM through cellular and animal experiments. Ultimately, this could pave the way for the development of P2X7-targeted therapies and more individualized approaches to managing T2DM.

## Conclusion

In conclusion, this study reported for the first time that three polymorphisms of P2X7(rs7958311, rs208294, rs11065464) are associated with the risk of T2DM and its complications. These findings enhance our understanding of the role P2X7 polymorphisms play in the pathogenesis of T2DM and may offer new therapeutic strategies for the prevention and personalized treatment of T2DM.

## Supporting information

S1 Table The primers and fluorescent probes sequence designed in this study. (DOCX)
