# Supplementary material for: Association of P2X7 polymorphisms on Type 2 diabetes mellitus susceptibility and diabetic complications
Source: PLoS One. 2025 Jan 27;20(1):e0318134. doi: 10.1371/journal.pone.0318134 (PMC11771865; doi:10.1371/journal.pone.0318134)
Supplement: S1 Table — (DOCX) [file pone.0318134.s001.docx]

| SNPs ID | Name | Sequence(5’-3’) |
| --- | --- | --- |
| rs1718119 | Forward Primer | CAGCGCTTGTCTGCATTCTC |
|  | Reverse Primer | AGTAAGTGTCGATGAGGAAGTCGAT |
|  | Probe1 | FAM-CCAGGCCGCTGTG-MGB |
|  | Probe2 | VIC-CCAGGCCACTGTGT-MGB |
| rs17525809 | Forward Primer | TCAGTTCTGTGCACACCAAGGT |
|  | Reverse Primer | TGCACCAACTTCTTCACTCCAT |
|  | Probe1 | FAM-TGAAAGAGGAGATCGCGGA-MGB |
|  | Probe2 | VIC-AAAGAGGAGATCGTGGA-MGB |
| rs2230911 | Forward Primer | CCAGGCCGCTGTGTTCAT |
|  | Reverse Primer | AGCACTTGCACCAGGGATAAA |
|  | Probe1 | FAM-CTCATCGACAGTTACT-MGB |
|  | Probe2 | VIC-TCATCGACACTTACTC-MGB |
| rs7958311 | Forward Primer | CAACCTAGACCGTTGGTTCCAT |
|  | Reverse Primer | AGGACACGTTGGTGGTCTTGT |
|  | Probe1 | FAM-ACTGCCATCCCAAA-MGB |
|  | Probe2 | VIC-ACTGCCGTCCCAAAT-MGB |
| rs208294 | Forward Primer | CGATGCTTTGACCCCTATAGGA |
|  | Reverse Primer | AGGCAGAGACTTCACAGGTCTTCT |
|  | Probe1 | FAM-AAGGTGTGTAGTGTATGAA-MGB |
|  | Probe2 | VIC-AAGGTGTGTAGTGCATGA-MGB |
| rs11065464 | Forward Primer | CAACACATCCATCACCACGTAGT |
|  | Reverse Primer | CCGTATCTGGGACTTGTATTTTACC |
|  | Probe1 | FAM-TATTTCATACGTAAATACG-MGB |
|  | Probe2 | VIC-ATTTCATACGTAACTACGCA-MGB |
